# The Changing Spectrum of Cognitive Impairment in People with HIV; Establishment and Updates of the International HIV-Cognition Working Group

**DOI:** 10.1007/s11904-026-00779-y

**Published:** 2026-04-09

**Authors:** Sam Nightingale

**Affiliations:** Neuroscience Institute, University of Cape Town, Cape Town, South Africa

## Abstract

With the widespread availability of effective antiretroviral therapy, the spectrum of cognitive impairment in people with HIV has changed from a subcortical dementia to milder impairment. The 2007 criteria for HIV-associated neurocognitive disorders (HAND) provided a methodological approach to classify milder and asymptomatic forms. Research using these criteria found that around 45% of people with HIV had HAND. This does not align with clinical observations that people with HIV supressed on modern treatment generally have good clinical outcomes, including cognitively. HAND criteria have several limitations which may account for an overestimation of prevalence including a high false classification rate, inadequate mitigation of psychosocial confounds to testing of cognitive performance, and the attribution to HIV of multifactorial causes of brain injury. This overestimation can hinder neuro-HIV research, increase anxiety around health outcomes, and worsen stigma and discrimination towards people with HIV. In response an International HIV-Cognition Working Group was established. The group proposed six consensus recommendations towards a new approach, including that HIV-associated brain injury (HABI) - which can be active or legacy - is conceptually separated from other cases of brain injury in people with HIV. The recommendations are aimed at harmonising clinical and research terminology, destigmatising terminology, and providing more accurate information on prevalence and prognosis. Cognitive impairment remains an important complication of HIV, particularly as the population ages, requiring a nuanced approach to classification and management.

## Commentary

From early in the HIV epidemic it was evident that HIV can affect the brain. Prior to antiretroviral therapy (ART) it was reported that one in six with advanced HIV had a moderate to severe cognitive impairment prior to death, which was typically of a subcortical phenotype with psychomotor slowing [1]. The nomenclature for this syndrome changed over the years: AIDS-dementia complex, HIV-encephalopathy and HIV-associated dementia all refer to a major neurocognitive disorder caused by HIV. This AIDS defining condition is now rarely seen, typically restricted to those with late presentations or failing antiretroviral (ART) treatment [2].

As ART coverage broadened and prognosis improved, milder forms of cognitive impairment persisted. Some people with HIV started ART with advanced disease at a point where some degree of irreversible brain injury may have already occurred, termed the legacy effect [3]. Others had incomplete response to ART due to drug resistance or incomplete adherence. In addition, some questioned whether there might be an ongoing process of brain injury in those with suppressed HIV RNA on ART. There was biological plausibility for this from several sources. ART penetration into the central nervous system (CNS) is generally lower than peripherally and cerebrospinal fluid (CSF) levels of several antiretroviral drugs are a fraction of that in plasma [4]. Indeed, in a minority of people with HIV, HIV can be found in the CSF despite being supressed in the plasma; a phenomenon termed CSF HIV RNA escape. Some cases of CSF HIV RNA escape are driven by compartmentalised virus in the CNS, and can lead to severe neurological disorders including rapidly progressive cognitive impairment, with impressive clinical improvements observed following change of ART directed at the resistance pattern of virus in the CSF [5]. These florid cases are uncommon, but CSF HIV RNA escape at lower levels has been observed in around 2–5% of apparently asymptomatic individuals raising the possibility that the CNS is not being adequately protected on a larger scale [6–8]. Even in those with supressed HIV RNA in plasma and CSF, neuroinflammatory biomarkers can be raised [9–11].

It was on this background that criteria for HIV-associated neurocognitive disorders (HAND) were established [12]. These criteria are often referred to as the *Frascati* criteria after the Italian town in which they were developed by a working group formed by the US National Institute of Mental Health and National Institute of Neurological Diseases and Stroke. HAND criteria included HIV-associated dementia at the most severe end, and established two new categories of milder cognitive impairment in people with HIV: mild neurocognitive disorder (MND) and asymptomatic neurocognitive impairment (ANI). HAND criteria provided a statistical approach to compare the cognitive performance of person with HIV to normative control data from people without HIV to determine a cut off for HAND, with the presence or absence of functional limitation (below the threshold for dementia) subclassifying HAND into MND or ANI.

HAND criteria, published in 2007, operationalised and harmonised the interpretation of cognitive testing in research, facilitating a large number of studies of cognition in people with HIV around the world. In 2021 a meta-analysis of HAND studies combined data from 123 studies in 32 countries involving 35,513 participants [13]. It found that 42.6% (95% CI: 39.7–45.5) of people with HIV had HAND, mostly ANI, representing over 16 million people globally. Rates in Africa were higher still with 72% of HAND cases being in sub-Saharan Africa. Based on these figures HAND would represent one of the leading causes of young onset cognitive impairment globally, and a major limitation of ART.

However, concern was raised by several authors that these criteria might overestimate the prevalence of cognitive impairment in people with HIV and controversy developed around the HAND criteria [14–20]. HAND prevalence figures did not seem to match clinical observations that people on suppressive ART appeared to have largely preserved cognition, and cognitive impairment was a relatively uncommon diagnosis in HIV clinics [21, 22]. Perhaps this was due to the hidden nature of asymptomatic deficits, however HAND was described as a progressive condition with ANI frequently progressing on to MND [23, 24]. The hospital I work in serves an area with HIV prevalence above the national South African average of 14%, however a mass epidemic of symptomatic cognitive impairment has not been apparent. Of course cognitive symptoms can be underreported due to lack of insight and potential stigma associated with cognitive difficulties, hence this observation alone does not exclude that mild cognitive impairment is common amongst people with HIV, however it led some to examine the HAND criteria more closely.

The HAND criteria have a number limitations which may explain some of this discrepancy [20]. Firstly, the statistical approach underlying the HAND criteria has a high false positive rate. HAND requires that performance on at least two cognitive domains falls at least 1SD below the normative population of people without HIV. In a normally distributed population this includes around 16% of cognitively unimpaired people. Given that only 2 cognitive domains of the 6 or 7 typically measured need to fall outside 1SD, this corresponds to a false positive rate of around 26% [19]. The rate varies depending on how many domains are measured and how the statistics are applied, with some reporting false positive rates up to 74%.^18^ On surveying the literature we found 20 different ways the statistical approach had been applied; when we applied each method to a single South African cohort of people with HIV, prevalence of cognitive impairment varied from 20% to 97% [25].

Secondly, minimum criteria for HAND (i.e. ANI) are met based on low performance on cognitive testing alone. However performance on cognitive testing is not an objective measure of neuronal integrity as it is strongly influenced by educational, cultural and socioeconomic differences. This is a widely accepted phenomenon in neuropsychology which is mitigated in the HAND criteria by the suggestion to control for the demographic variables of age, sex, years of education and ethnicity [12]. However, these variables are unlikely to account for the complexity of psychosocial variability and its effect on cognitive performance. People with HIV can have different psychosocial backgrounds to the general population related to the vulnerabilities associated with HIV acquisition [26] Recent data from our group has demonstrated that controlling for standard demographic variables alone (i.e. age, sex and years of education) incompletely mitigates these psychosocial confounds to cognitive testing [27].

Thirdly, not all cognitive impairment in people with HIV is necessarily caused by HIV. HAND addressed this by categorising comorbidities as ‘contributing’ or ‘confounding’ [12]. Those with a severe comorbidity such as Alzheimer’s disease would be considered confounded and not classified as HAND. Those with milder comorbid conditions that could affect the brain, for example head injury or cerebrovascular disease, would be categorised as ‘HAND with contributing comorbidity’. This implies that HIV is causing the cognitive disorder, at least in part, even in the absence of evidence of that being the case in a given individual. Comorbidities can be common in populations of people with HIV and can impact rates of cognitive impairment dramatically. For example in the CNS HIV Antiretroviral Therapy Effects Research (CHARTER) cohort, 40% of those with minimal comorbidities were classified as having HAND compared with 59% and 83% of those with moderate or severe comorbidities respectively [28]. Even those in the group termed minimal comorbidities, potential confounders were common; 71% of this group had a history of drug use and 16% had current depression or psychotic disorder. It is not clear to whether HIV contributed to brain injury in all these people, or whether the brain injury was caused by these other factors in a person that happened to have HIV. A combination of the two might be true, with HIV creating a vulnerability to brain injury from other causes.

Data supports that rates of cognitive impairment caused by HIV may be lower than reported in HAND studies. In the Multicenter AIDS Cohort Study of 2904 men in the US (1531 living with HIV), HIV serostatus was not associated with cognitive impairment [29], and the rate of decline over time was no different to a matched HIV-negative population [3]. Similarly, for 1521 women in the US (1019 living with HIV), the effect size for HIV status on cognition was very small, accounting for only 0.05–0.09 SD units, far less than the impact of education, age, race, income, and reading level [30]. In the CHARTER cohort of 402 people with HIV in the US followed over 12 years, cognitive decline was unrelated to HIV disease or treatment characteristics but was significantly predicted by the presence of comorbid conditions [31]. In Italy, HAND rates in 1424 people with HIV declined over an 11 year period from 2009 to 2020. By the end of the study HIV-associated dementia and MND rates were just 0.4% and 1.1% respectively, and ANI rates fell to a level similar to the false positive rate of the test [2, 19]. In South Africa our CONNECT study of 273 people (178 living with HIV) demonstrated similar cognitive performance in people with and without HIV once psychosocial factors and the neurotoxic effect of efavirenz were accounted for [27, 32].

Although most agree HAND criteria need to be updated [33], there remains some disagreement over the extent to which HAND misrepresents cognitive impairment in people with HIV and full consensus on the matter is yet to be attained [34]. Despite concerns around HAND data, many scientific publications and presentations continue to assert that almost half of people living with HIV have a neurocognitive disorder despite access to modern ART. If this statement is as inaccurate as I have described, there are a number of important consequences for people with HIV. Firstly, it hinders scientific progress in the field. Research aiming to examine mechanisms underlying cognitive impairment in people with HIV is reduced by such a broad phenotype. Indeed, no clear mechanisms for HAND have been identified [35], and all HAND treatment studies have so far been negative [36–39]. Secondly, it is off message for ART effectiveness in an era where we are trying to emphasise the health benefits of ART and normalise living with HIV. Thirdly, and perhaps most importantly, there are implications for stigma and discrimination. Cognitive impairment carries its own stigma and there have been examples of discrimination towards people with HIV due to the misrepresentation of this population being at high risk for cognitive decline. As an example, until recently pilots living with HIV in the UK were not able to work, in part due to concerns around their cognition. This was challenged by a pilot living with HIV who had been prevented from working despite being virally suppressed and demonstrating unimpaired cognitive functioning on the standard testing all pilots must routinely undergo. Fortunately this discriminatory ruling was overturned, allowing him to work alongside his HIV-negative colleagues.

In response to these concerns we established an International HIV-Cognition Working Group aiming to establish expert consensus on a way forward for the field. This working group comprised 20 clinicians, academics and HIV-community representatives from around the globe, with around half from low-and middle-income countries experiencing high HIV prevalence. Together we established 6 consensus recommendations towards a new approach [40]. The first recommendation coined the term ‘HIV-associated brain injury’ (HABI) to represent the direct effect of HIV on the brain. We suggested that HABI should be conceptually separated from brain injury due to other causes (such as cerebrovascular disease or traumatic brain injury), although multiple mechanisms may coexist. The second was that HABI should be further differentiated into legacy (i.e. due to pre-ART CNS damage which is non-progressive), or active (leading to clinical or radiological progression). Importantly, it is not clear whether active HABI occurs in the presence of sustained HIV suppression in plasma and CSF, and defining active HABI as a distinct entity is intended to facilitate research into that important question. The third recommendation was that those with cognitive performance occurring below a certain threshold should be termed as having ‘low cognitive performance’, rather than diagnosed with ANI or HAND. This recognises that, although there may be benefit defining a group at the lower end of the cognitive spectrum in research studies, the language should be destigmatised such that they are not labelled with a disease. The forth recommendation is that rates of low cognitive performance should be interpreted in the context of the false classification rate underlying whichever statistical methodology was applied. For example a low cognitive performance prevalence of 30% is interpreted very differently in the knowledge that the false classification rate is 26%. Fifth we propose that a classification of cognitive impairment be made if at least two of the following are present: low cognitive performance, cognitive symptoms, and objective evidence of brain injury (i.e. abnormality on neurological investigations). And lastly that cognitive symptoms should refer to any change in cognition that has been noticed by the individual or an observer, whether or not this change has an impact on daily functioning (as was the case in HAND criteria), recognising the importance of milder symptoms in the modern era.

Our approach is intended as a pragmatic reframing of the issue to harmonise research terminology with clinical diagnosis. In contrast HAND was a research criteria never intended for clinical use. Our approach was adopted by the European AIDS Clinical Society in 2023 [41], and it is hoped that further discussion and refinement will lead to a broader consensus and agreement on new criteria going forward.

Cognitive impairment remains an important complication of HIV, with devastating consequences for those affected. It is a particularly important area given the ageing population of people with HIV globally. It is not known how HIV interacts with the ageing process and whether subclinical legacy HABI may lower cognitive reserve increasing vulnerability to cognitive ageing. These research questions require a robust and nuanced approach given the complex and multifactorial nature of brain health in people with HIV.

## Data Availability

No datasets were generated or analysed during the current study.
